# Personals

**Published:** 1915-11

**Authors:** 


### Personals.

An interesting and impressive memoriam was recently held at Kyle and many beautiful tributes paid to the late Dr. J. M. Pound, who died at that place on October 20, 1914.

Dr. Z. J. Spruiell of Jewett recently made a valuable and masterly address on the subject of hygiene, diet, etc., before the social meeting in the high school auditorium at Centerville.

Dr. and Mrs. W. N. Walker of Celina are rejoicing over the arrival of a new heir at their house. The doctor is all smiles now, as this is the first son. Mother and child reported getting along nicely.

Dr. Jefferson D. Gibson of Denver, president of the American Association of Clinical Research, declared in an address recently delivered in Philadelphia, that within ten years medical science will probably have succeeded in practically eliminating death from tuberculosis.

